# Does intrauterine saline infusion by intrauterine insemination (IUI) catheter as endometrial injury during IVF cycles improve pregnancy outcomes among patients with recurrent implantation failure?: An RCT

**Published:** 2016-09

**Authors:** Saghar Salehpour, Marzieh Zamaniyan, Nasrin Saharkhiz, Shahrzad Zadeh modares, Sedighe Hosieni, Samira Seif, Narges Malih, Parinaz Rezapoor, Mohammad-Reza Sohrabi

**Affiliations:** 1 *Preventive Gynecology Research Center (PGRC), Shahid Beheshti University of Medical Sciences, Tehran, Iran.*; 2 *Infertility Center, Department of Obstetrics and Gynecology, Mazandaran University of Medical Sciences, Sari, Iran.*; 3 *Department of Veterinary Theriogenology and Obstetric, Tehran University of Medical Sciences, Tehran, Iran.*; 4 *Department of Health and Community Medicine, Faculty of Medicine, Shahid Beheshti University of Medical Sciences, Tehran, Iran.*

**Keywords:** *Artificial Insemination*, *Embryo implantation*, *Embryo Transfer*, *Fertilization in Vitro*, *Endometrium*, *Injuries*, *Pregnancy*

## Abstract

**Background:**

Recurrent implantation failure is one of the most issues in IVF cycles. Some researchers found that beneficial effects of endometrial Scratching in women with recurrent implantation failure, while some authors demonstrated contrary results

**Objective:**

The present study aimed to investigate the effect of intrauterine. Saline infusion as a form of endometrial injury, during fresh in vitro fertilization-embryo transfer cycle, among patients with recurrent implantation failure.

**Materials and Methods:**

In this clinical trial study 63 women undergoing assisted reproductive technology were divided into two groups either local endometrial injury by intrauterine saline infusion during day 3-5 of the ongoing controlled ovarian stimulation cycle, or IVF protocol performed without any other intervention in Taleghani Hospital, Tehran, Iran. The main outcome measure was clinical pregnancy rates.

**Results:**

Patients who received intra uterine saline infusion (n=20), had significantly lower clinical pregnancy numbers (1 vs. 9, p<0.05) and implantation rates (4.7% vs. 41.6%, p<0.05), compared to controls (n=39). However, there was no significant difference in miscarriage rates (9.4% vs. 8.7%, p>0.05) and multiple pregnancy numbers (1 vs. 3, p>0.05) between groups.

**Conclusion:**

When intrauterine saline infusion as a form of endometrial injury is performed during the ongoing IVF cycles it has negative effect on reproductive outcomes among patients with recurrent implantation failure.

## Introduction

In assisted reproductive technology (ART), embryo culture conditions and transfer methods have improved over recent decades, but the pregnancy and delivery rate has not increased. It is predicted that about 10% of women who undergo in vitro fertilization (IVF) treatment will face specific problems (1). Recurrent implantation failure (RIF) is a main cause of repeated IVF failure (2). There are some differences in definition of RIF. One favorable definition of RIF is having at least three unsuccessful IVF with transfer of good-quality embryos without a successful implantation or alternatively another definition is when women fail to conceive from IVF after cumulative transfers exceeding 10 high-quality embryos (3). RIF is the most important dilemma that physicians and patients frequently encountered with it. Among the different causes of RIF, uterine factors, including thin endometrium, poor endometrial receptivity and immunological factors have received more attention (4). Numerous signaling molecules, including cytokines, growth factors and interleukins are secreted from the endometrium, which could modify endometrial receptivity and dysregulation of these factors, can result in RIF (5-7).

Several studies have indicated that local endometrial injury (EI) caused by hysteroscopy, scratching or biopsies during IVF cycles are associated with local inflammatory response which subsequently leads to improved implantation and pregnancy among women with RIF (8-20). 

There is also some evidence showing that EI in IVF cycles immediately after oocyte retrieval does not have any deleterious effects on embryo implantation, but adverse effects could be observed when the biopsy is performed immediately before embryo transfer (21, 22). Some other studies have suggested that EI during the ongoing stimulation cycle could cause better pregnancy outcomes (9, 18, 23). Nevertheless, one study reported that EI on the day of oocyte retrieval had negative effects on clinical outcomes (24). There is also some evidence indicating that hysteroscopy per se in early follicular phase of proceeding cycles of IVF causes a higher pregnancy rate in experimental patients compared to the control group (14-16, 20). 

The present study is the first study to investigate the intrauterine saline infusion as endometrial injury among RIF patients, which seems to act like hysteroscopy alone. In the present study, we assumed that, if a local EI can be a helpful factor for increased pregnancy outcomes, a local mechanical injury by intrauterine saline infusion and subsequently suctioning it at day 3-5 of ovarian stimulation during ongoing IVF cycle should produce similar effects with lower costs and complications compared to hysteroscopy or biopsy.

## Materials and methods

The present study was approved by the institutional ethics committee review board of Shahid Beheshti University of Medical Science, Tehran, Iran. All women gave written informed consent before the start of the study. 

**Patients**

A total of 63 women with RIF were recruited in this clinical trial study which was done in Taleghani Hospital, Tehran, Iran, during April to December 2014. Finally, 59 women, who had failed three or more in vitro fertilization-embryo transfer (IVT-ET) cycles or took replacement of more than 10 embryos in previous failed IVF-ET cycles, met our criteria and recruited for the study. Women aged 20-40 years old with body mass index (BMI) ≤30 kg/m^2^ or ≥ 18 kg/m^2^, who underwent fresh intra cytoplasmic sperm injection-embryo transfer (ICSI-ET) cycles, and had normal endometrial thickening (7-12 mm) on the day of oocyte retrieval and did not have any visible endometrial pathology were included within the study. Women with poor ovarian reserve (defined as serum level or anti-Müllerian hormone (AMH) ≤1.1 ng/mL or antral follicular count (AFC) <5 or ≤3 oocytes were retrieved in previous IVF cycles), advanced endometriosis, pelvic or uterine adhesion, hypothyroidism and hyperprolactinemia, genital tuberculosis or two or less previous IVF failures were excluded (32). 

Work up of RIF, such as ruling out uterine pathology (synechia, sub mucosal myoma, polyp) using diagnostic hysteroscopy, an autoimmune serology test and karyotype of couples, was performed for all participants. Couples, in whom there was no evidence of pathology, received an offer to undergo intra uterine saline infusion during ongoing assisted reproduction cycles. Out of 59 participants 20 women agreed and underwent this procedure and the remaining 39 patients underwent regular IVF.

**Hyper stimulation protocol and saline infusion procedure**

All women underwent GnRH antagonist protocol as follow: controlled ovarian stimulation (COS) started using recombinant FSH (rFSH, Gonal-F; Merck Serono SA, Geneva, Switzerland) 225-400 IU/day for the first five days. Subsequently, the rFSH dose was adjusted along with ovarian response, every other day. During the COS in the intervention group, intrauterine saline infusion procedure was performed on day 3-5 of stimulation using a gynecologic examination table. 

No drug or anesthesia was used. Cusco’s speculum was inserted through the vagina in order to visualize the cervical orifice. After sterilization with Povidone iodine (10%) and full irrigation with normal saline, intrauterine insemination (IUI) catheter (Biorade, Berkeley, California) was introduced gently into the uterine cavity with a syringe and then 20ml of normal saline was introduced into the uterine cavity and aspirated immediately. Then follicular tracking and endometrial measurement were completed using a 7.5 MHZ transvaginal ultrasound transducer.

GnRH antagonist (Cetrotide 0.25 mg; Merck Serono SA) was started once the leading follicle reached 14-15 mm, and was continued until the day of hCG injection. A recombinant hCG (Ovitrelle; Merck Serono SA, Geneva, Switzerland, 250 μg) was injected to trigger final follicular maturation when three or more follicles reached a mean diameter of ≥17 mm (25). Oocyte retrieval was performed 36 hrs later under conscious sedation. Then each oocyte was denudated with hyaluronidase. 

Conventional Intra-Cytoplasmic Sperm Injection (ICSI) was performed for all patients. The embryo grading was based on Veeck’s classification (26). One to two good or top quality embryos were transferred 72-96 hr after oocyte retrieval. Luteal phase was supported by the administration of micronized progesterone (600mg/day, Cyclogest; Actavis; Barnstaple; UK), which was stopped if the pregnancy test (serum β-HCG level test performed on day 15 after embryo transfer), was negative or after 12 wks of pregnancy.

**Statistical analysis**

Based on an Altman’s nomogram by power of 65% and a Standardized Difference of 0.6 to detect significant differences between two study groups, with respect to the pregnancy rate according to the previous pilot report on endometrial biopsy for increasing the success rate in IVF cycles, 20 cycles of IVF were required for each group (9). 

We included 39 patients (about two times the number of case group) as the control group for achieving more reliable results. The statistical software package SPSS for windows, version 21.0 (SPSS, Armonk, NY: IBM Corp) was used for data analysis. T-test was used to compare the results between the groups. The proportions were compared using the  ^2^test. p˂0.05 were considered as significant.

## Results

**Figure 1:**
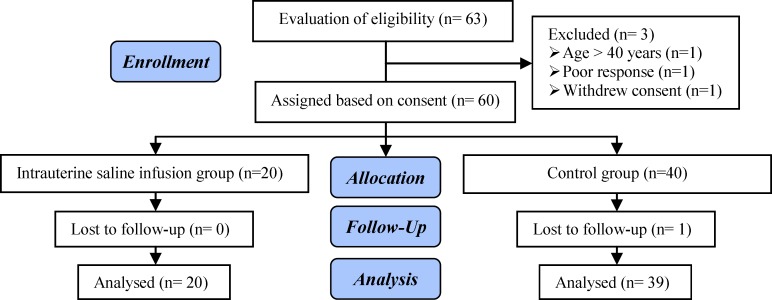
Flow chart of the eligible patients.

During the eight-month period (April to December 2014), a total of 63 patients with RIF were recruited. Of these, 59 women met our inclusion criteria.Finally, 20 women were assigned to the intervention group after agreeing to undergo the new method and 39 remaining patients were included in the control group (Figure 1). The mean±SD age among the women in the intrauterine saline infusion group was 37.7±5.63 years and in the control group it was 34.0±6.04 years (p=0.49). Baseline and clinical characteristics among RIF patients with or without the intrauterine saline infusion procedures are presented (Table I). 

Chemical (2 vs. 9, p=0.03), and clinical pregnancy numbers (1 vs. 9, p=0.01), as well as implantation rates (4.7% vs. 41.6%, p=0.0035), were significantly higher in control group compared to those in the saline infusion group. There was no significant difference in miscarriage rates (9.4% vs. 8.7%, p=0.403) and multiple pregnancy numbers (1 vs. 3, p=0.39) between two groups (Table II).

**Table I:** Baseline characteristics in RIF patients with and without the intrauterine saline infusion

**Variable**	**Saline infusion group** **(n= 20)**	**Control group** **(n= 39)**	**df**	**t**	**95% CI**	**p-value**
age (year)	37.7 ± 5.63	34.0 ± 6.04	40	2.03	0.02 to 7.3	0.49
BMI (kg/m^2^)	24.6 ± 3.1	25.9 ± 2.47	40	-1.48	-3.04 to-0.46	0.14
FSH (mIU/ml)	9.9±8.08	7.1± 2.59	40	1.59	-4.94 to 2	0.11
AMH (ng/ml)	3.0 ± 2.89	4.51 ± 7.00	40	-0.85	-1.47 to -1.71	0.39
Endometrial thickness on HCG day (mm)	7.1 ± 2	7.1 ± 0.49	40	-0.09	-0.91 to 0.82	0.14
Total number of previous failed cycles	2.8 ± 1.10	2.5 ± 0.94	40	1.18	-0.27 to 1.02	0.24

Data are presented as Mean ± SD.

Independent *t* test was used.

**Table II:** Comparison of clinical outcomes in RIF patients with and without the intrauterine saline infusion

**Variable**	**Saline infusion group** **(n= 20)**	**Control group** **(n= 39)**	**p-value**
Fertilization rate (%)*	73.4%	75.6%	0.24 ^a^
Patients with good and top quality embryos *	76.1%	74.3%	0.75 ^a^
Implantation rate (%)*	4.7%	41.6%	0.003 ^a^
Miscarriage rate (%) (before 24 weeks /total pregnancy) *	9.4%	8.7%	0.40 ^a^
Number of embryos transferred**	2.3 ± 0.74	2.3 ± 0.76	0.96^b^
Total number of oocyte retrieval **	10.7 ± 3.4	11.8 ± 3.05	0.24^b^
Total number of previous embryo transferred **	7.8 ± 4.48	8.4 ± 2.34	0.52^b^
Chemical pregnancy number	2	9	0.03 ^a^
Clinical pregnancy number	1	9	0.01 ^a^
Multiple pregnancy number	1	3	0.39 ^a^

* Data are presented as %.

** Data are presented as Mean±SD

a  ^2^ test

b Independent *t* test

## Discussion

In this present study, we investigated the effects of intrauterine saline infusion as endometrial injury on day 3-5 during ongoing ovarian stimulation in IVF cycles on pregnancy and implantation rates. Our objective was to introduce intrauterine saline infusion as a novel procedure to induce endometrial injury for improving implantation in IVF cycles for patients with RIF. 

Some animal studies have reported that oil injections into the uterine cavity result in uterine decidualization (27, 28). We assumed that if hysteroscopy without biopsy has been found in some studies to lead to increased pregnancy rate, a less invasive procedure like intrauterine saline infusion, which has no adverse effects or does not require anesthesia, may improve implantation in IVF cycles as well (14-16). Contrary to previous results, our study showed that chemical and clinical pregnancy and implantation rates were significantly lower in intervention compared with control group (9-11, 18, 20, 29). 

There are inconsistent clinical results concerning EI. Some studies have reported that endometrial injury causes better clinical outcomes, but other studies have reported similar results (9, 10, 12, 18, 20, 29, 30). In one meta-analysis, mechanical endometrial injury (by hysteroscopy or biopsy or scratching) carried out as ovarian stimulation during IVF cycle was found to increase clinical pregnancy rates in women with RIF. The authors concluded that this procedure may yield iatrogenic effects and it should not be done routinely in clinical practice due to insufficient evidence (10). 

In a recent Cochrane's review by Nastri *et al* it was reported that EI in women undergoing ART between seven days of preceding cycle and the day of ET cycle versus no intervention was associated with increased live birth rate (LBR) in women with more than two previous embryo transfers. These researchers found no evidence regarding the effect of EI on miscarriage, multiple pregnancies or bleeding except mild pain (11). In another study, endometrial scratching by Pipple twice on day 21 or 22 immediately prior to IVF cycle, had similar LBR compared to the control group, but in a subgroup of patients with two or more previous IVF failures, it improved LBR. These researchers noted that this procedure should be used in luteal phase of preceding IVF cycle to stimulate gene expression for implantation, since when this procedure was used in the pre ovulatory phase of the ongoing IVF cycle; it could disrupt the endometrial proliferation and embryo implantation (30). 

In contrast Baum *et al* reported that local injury to the endometrium by Pipple twice in follicular and luteal phase of preceding IVF cycle did not have any benefits in high-order RIF patients and that clinical outcomes in the intervention group were significantly lower than those in the control group. They suggested that these lower clinical results might be due to intrinsic embryonic factors in high-order RIF patients and did not correlate with endometrial receptivity (31). To our knowledge the present study is the only study aiming to incorporate an intra-uterine saline infusion procedure as local endometrial injury to improve pregnancy outcomes. 

This experimental study included only 20 patients undergoing this procedure, but the sample size was insufficient to demonstrate definitive unfavorable results. Our results suggested that patients with RIF get no advantage from injury to the endometrium as intra uterine saline infusion in ongoing cycle.

## Conclusion

When intrauterine saline infusion as a form of endometrial injury is performed during the ongoing IVF cycles it has negative effect on reproductive outcomes among patients with recurrent implantation failure.

## Note

***Registration ID in IRCT: IRCT2014120219426N3***
